# An Amazon stingless bee foraging activity predicted using recurrent artificial neural networks and attribute selection

**DOI:** 10.1038/s41598-019-56352-8

**Published:** 2020-01-08

**Authors:** Pedro A. B. Gomes, Yoshihiko Suhara, Patrícia Nunes-Silva, Luciano Costa, Helder Arruda, Giorgio Venturieri, Vera Lucia Imperatriz-Fonseca, Alex Pentland, Paulo de Souza, Gustavo Pessin

**Affiliations:** 1 Institute of Exact and Natural Sciences, Federal University of Pará, Belém, PA 66075-110 Brazil; 2 Media Lab, Massachusetts Institute of Technology, Cambridge, MA 02139 United States; 3 Instituto Tecnológico Vale, Belém, PA 66055-090 Brazil; 4 PPG Biologia, Universidade do Vale do Rio dos Sinos, São Leopoldo, RS 93022-750 Brazil; 5 Polytechnic School, Universidade do Vale do Rio dos Sinos, São Leopoldo, RS 93022-750 Brazil; 6 Embrapa Amazônia Oriental, Belém, PA 66095-903 Brazil; 7 Nativo, Brisbane, QLD 4012 Australia; 8 Instituto de Biociências, Universidade de São Paulo, São Paulo, SP 05508-090 Brazil; 9 Data61, Commonwealth Scientific and Industrial Research Organisation, Sandy Bay, TAS 7005 Australia; 10 School of Information Communication Technology, Griffith University, Gold Coast, QLD 4222 Australia; 11 Universidade Federal de Ouro Preto, Ouro Preto, MG 35400-000 Brazil; 12 Instituto Tecnológico Vale, Ouro Preto, MG 35400-000 Brazil

**Keywords:** Ecological modelling, Environmental sciences

## Abstract

Bees play a key role in pollination of crops and in diverse ecosystems. There have been multiple reports in recent years illustrating bee population declines worldwide. The search for more accurate forecast models can aid both in the understanding of the regular behavior and the adverse situations that may occur with the bees. It also may lead to better management and utilization of bees as pollinators. We address an investigation with Recurrent Neural Networks in the task of forecasting bees’ level of activity taking into account previous values of level of activity and environmental data such as temperature, solar irradiance and barometric pressure. We also show how different input time windows, algorithms of attribute selection and correlation analysis can help improve the accuracy of our model.

## Introduction

Bees, for dietary requirements, forage on nectar and pollen produced by plants; in doing so, plants are passively pollinated. Bees’ total requirements on plants for nutrition means that large scale foraging results is highly efficient pollinators. An estimated 35% of human food production is dependent on bees’ pollination services^1^. Brazilian stingless bees are important pollinators. In Amazon, Melipona bees are well represented, they produce honey as an attractive way for rearing by traditional people^2,3^. Worldwide, honeybee population declines have been reported since the 1960s^1^. The decline in pollinator numbers has ecological and agricultural, and subsequent economic consequences^4^. Factors responsible for colony declines have not been solely implicated, but include (i) parasites, (ii) pesticides, (iii) weather changes, (iv) monoculture farming, and (v) mismanagement of beehives^5^.

In order to investigate these risk factors and safeguard pollinators’ health, we argue that predictive models can aid in the identification of behavior patterns. The predictive model can aid in the following manners: (i) Monitoring the activity level of bees when their hives are managed for pollination may indicate when they are most visiting the crop. It can be used to avoid applying pesticides during peak activity or to evaluate if their activity matches time-related pollination requirements of the crop. (ii) When the current behavior of the bees does not match the predicted one, it may indicate that there is something different around the hive. It may trig an action from the farmer to verify the hive environment. (iii) Determining the environmental variables that influence bees’ behavior.

Taking into account the above-mentioned points, we address an investigation related to forecasting of bees behavior. We employ Recurrent Neural Networks (RNNs)^6,7^ and perform an investigation with several RNNs architectures; we also take into account different weather variables aiming to understand the impact of each weather variable on the level of activity. Therefore, the contributions of this paper are as follow: (i) after exploiting several RNN architectures, we show which perform best at forecasting bee behavior, (ii) we show how different input size windows impact on the accuracy of the forecast, and (iii) we show how algorithms of attribute selection and correlation analysis can help in improving the accuracy of the forecast. Hence, this work extends the work by Gomes and collaborators^8^ by presenting a detailed new investigation on the forecast problem with RNN using environmental data as a descriptor along with the bees’ activities. Furthermore, we exploit attribute selection techniques, aiming to find the best environmental features to increase the forecast accuracy.

Animal behavior science is an expanding field, employing technology and analytical improvements to further develop understanding of animal behavior. Schultz and colleagues^9^ present mechanisms of flight guidance in honeybee swarms. They argue that when a honeybee swarm takes off aiming to fly to a new location for its site, less than 5% of the bees in the swarm have visited the site. Schwager *et al*.^10^ employed clustering techniques to understand different behaviors in groups of cows. Schaerf and colleagues^11^ present how the characterization of the interactions can aid in the understanding of emergent phenomenon.

Improvements in the understanding of bee behavior are also sought by Chena *et al*.^12^, where it is employed an image-based tracking system. Tu *et al*.^13^ also exploit a computer vision system to analyze the behavior of honeybees. Gil-Lebrero *et al*.^14^ proposes a remote monitoring system to record temperature and humidity of hives, with very low interference in the regular behavior of the bees. In our study, we employ Radio-Frequency IDentification (RFID) tags. The advantage about RFID is that we can observe the behavior of individual insects and it allows us to avoid reading other insects (ants, wasps, or other species of bees) that may be entering the hive (for spoliation for example). It also presents good results for any light and weather condition. Arruda and collaborators^15^ and Gama and collaborators^16^ also employ the same RFID technology we use in our research. Arruda *et al*.^15^ present a methodology to identify different species of bees by its behavior. In their investigation, the Random Forest^17^ algorithm presented the best results for the classification. Gama *et al*.^16^ uses a time series of RFID collected data aiming to validate a methodology to analyze behavioral anomalies where a Local Outlier Factor^18^ algorithm is investigated for the anomaly detection.

Gated Recurrent Unit (GRU)^6^ and Long Short-Term Memory (LSTM)^7^ recurrent unit structure are investigated in our work. Martens and Sutskever^19^ and Chung and collaborators^20^ present evaluations of recurrent neural networks in other domains, where data appears sequentially. Chung and collaborators^20^ highlight that “The results clearly indicate the advantages of the gating units over the more traditional recurrent units. Convergence is often faster, and the final solutions tend to be better. However, the results are not conclusive in comparing the LSTM and the GRU, which suggests that the choice of the type of gated recurrent unit may depend heavily on the dataset and corresponding task”. Furthermore, Jozefowicz and colleagues^21^ showed that for a great class of problems, GRU outperformed LSTM. Their study is also corroborated by Carvalho and colleagues^22^, were GRU units showed lower dispersion than LSTM on the results.

Another factor which influences the capabilities of the neural networks is its number of hidden layers. In an intend to better understand recurrent neural networks, Karpathy, Johnson, and Fei-Fei^23^ performed several evaluations that allowed them to argue that results are improved by the use of an at least two-level architecture. In their evaluations, the use of a three-level architecture did not improve the results consistently, as they show that results from a three-level or two-level were somehow similar. Besides, they show that LSTM and GRU cells also performed with slight results differences, but outperforming the not-gated RNN. The massive exploration of Recurrent Neural Networks performed by Britz and colleagues^24^ showed that results from LSTM networks outperform GRU networks. Britz and colleagues also point out that, related to training speed, both architectures presented similar results, as the computational bottleneck in their structure was the softmax transfer function. In summary, the best architecture depends on the case in study.

Machine learning models might be improved by selecting the best features in a given context. Altmann *et al*.^25^ suggest a method called Permutation Feature Importance (PFI) where the features are evaluated and the best ones receive a greater score. This technique is commonly used in Random Forests, as described by Breiman^26^. Considering RNN and attribute selection mechanisms, Suhara *et al*.^27^ showed a problem where the PFI allowed obtaining better results, by removing lower score features. In our work we also exploit the Permutation Feature Importance score and extend it by a correlation analysis. We evaluate several topologies of LSTM and GRU neural networks to forecast bees’ level of activities, taking into account environmental data.

## Methods

### Data Collection

**Figure 1:**
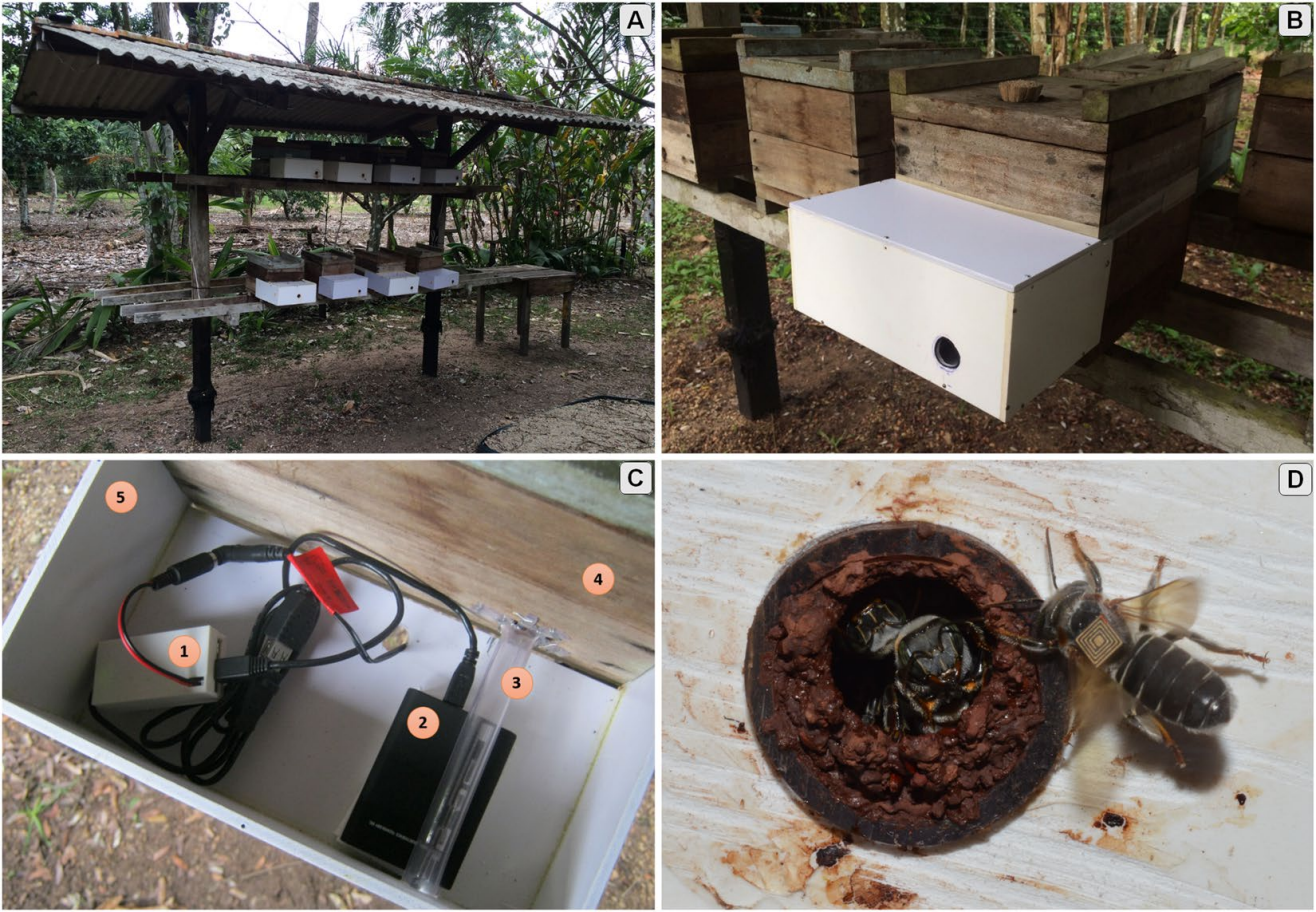
The system environment. (**a**) The eight *M*. *fasciculata* hives employed in this study. (**b**) Frontal view of the adapted hive entrance: it contains a PVC box for storing electronic items. (**c**) Electronic system details: (1) Intel Edison TM for RFID reader control and data storage, (2) USB RFID reader, (3) Transparent hose where the bees pass upon the RFID reader, (4) Hive of *M*. *fasciculata*, and (5) PVC box. (**d**) Tagged *M*. *fasciculata* at the hive entrance.

Activities from 1280 bees were collected from August 1st to 31st, 2015. The bees were tagged with UHF RFID tags (Hitachi Chemical, Tokyo), as shown in Fig. 1(d). The tags were glued to the thorax of the bees using Super Glue (Henkel Corp, Düsseldorf). The tagged bees were evenly distributed between 8 hives, as shown in Fig. 1(a). In this 4-week data collection, more than 127,000 activities we recorded. The RFID tags allow us to track bees’ behavior, which is used to advance baseline knowledge about this species. We choose to study the *Melipona fasciculata* because it is a native social bee of the Amazon region, very important for pollination and honey production.

Figure 1 presents the system environment and Fig. 1(b) presents a frontal view of the adapted hive entrance, containing a PVC box for storing electronic items. Figure 1(c) shows electronic system details, containing a Intel Edison TM for RFID reader control and data storage, and the USB RFID reader. Every occasion a tagged bee pass by the RFID reader, it records a data point with a timestamp and the individual bees’ ID number. Along with bees’ activity, we also collected data from temperature (°C), barometric pressure (hPa) and solar irradiance (kJ/m^2^). Detailed hardware and software system design can be found in the work by de Souza and colleagues^28^.

**Figure 2:**
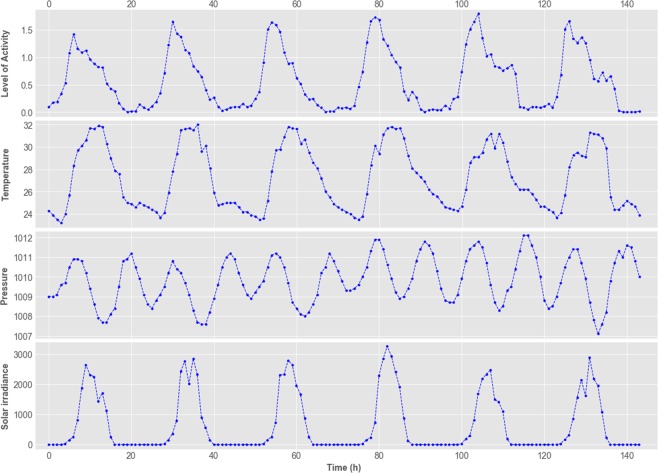
Section of the time series employed in this research, from August 7th to 12th, 2015. It presents hourly values of bees’ level of activity, temperature (°C), barometric pressure (hPa) and solar irradiance (kJ/m^2^).

We define the bees’ level of activity as the hourly total number of bees’ movements, divided by the number of tagged bees. The level of activity, per bee per hour, ranges from 0.0 (no bee performing any activity) to approximately 2.0 (two movements per hour). During our data collection phase, there were between 240 and 320 tagged bees on average, per day. Figure 2 shows a section of the data employed in this research, where the time series of activity level and weather can be observed.

### The Model

Multi-layer Perceptron Artificial Neural Networks are a machine learning method engineered as an analogy to the brain’s behavior^29,30^. Simple processing units, called neurons, linked in a network, are responsible for calculating mathematical functions that allow fitting inputs to outputs^31^. Depending on the problem we want to solve, different architectures (or topologies) can be exploited in the search for the best fit. Linearly separable problems often can be solved with one layer of neurons, however, non-linear problems usually need more layers to be able to perform best fits. Neural Networks have been applied in many contexts, such as forecasting of drylands^32^, classification of human electroencephalogram^33^, monitoring of memory^34^, robotics and computer vision^35^.

Recurrent Neural Networks extends regular Neural Networks by adding the capability of recurrences within the neurons. This recurrence allows the network to handle variable-length sequences^36^. In doing so, the Recurrent Neural Networks present the ability to store internal memory and to deal more naturally with dynamic temporal behavior^37–39^. In its usual form, the recurrence is represented by ${h}^{(t)}=f({h}^{(t-1)},{x}^{(t)};\theta )$, where *h* is the hidden state at time *t*. ${h}^{(t-1)}$ represents the previous hidden state. ${x}^{(t)}$ is the current input vector and *θ* is the set of shared parameters through time. As mentioned by Gomes *et al*.^8^, originally, RNNs were difficult to train due to the problem of the vanishing gradient. It is also mentioned in the work by Chung *et al*.^20^. Huang *et al*.^40^ describe this phenomenon in the following way: “as the gradient information is back-propagated, repeated multiplication with small weights renders the gradient information ineffectively small in earlier layers”. Hence, to overcome this problem, some methods have been proposed, as the clipped gradient presented by Chung and colleagues^20^ and the use of activation function with gated units. Gated units are able to monitor the quantity of data that enters the unit, the quantity of data that is stored and the quantity of data that is forwarded to the next units. The two more effective types of gated unit are the Long Short-Term Memory (LSTM)^7^ and the Gated Recurrent Units (GRU)^6^.

For the RNN deployment, we use Keras (https://keras.io) with Theano (http://deeplearning.net/software/theano) backend. Scikit-Learn (https://scikit-learn.org/stable) was also used to allow getting metrics and methods for normalization. The RNN was built on Python 3.7.

### Exploiting RNN Topologies

**Figure 3:**
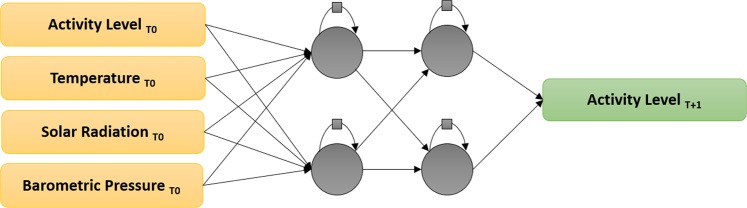
One of the developed topologies: it consists of 4 neurons organized in two hidden layers. In this figure, we show as inputs: Activity Level, Temperature, Solar Irradiance and Barometric Pressure. The output is the forecast of Activity Level at t + 1. Evaluated hidden layers are LSTM and GRU.

In order to find the most suitable RNN topology to forecast the activity level of bees, we initially investigate eight different recurrent neural networks, considering four topologies (with different number of neurons and layers) and different gated units (GRU and LSTM). The designed topologies were built with: two hidden layers with two recurrent units in each layer (2X2), two hidden layers with five recurrent units in each layer (2x5), five hidden layers with two recurrent units each (5x2), and five hidden layers with five recurrent units in each layer (5x5). Hence, the eight models designed to the investigation are: {GRU2x2, GRU2x5, GRU5x2, GRU5x5} and {LSTM2x2, LSTM2x5, LSTM5x2, LSTM5x5}. Figure 3 depicts one of the proposed architectures (specifically 2x2).

Related to training and testing each architecture, we employ a hold-out method. We evaluate the model using the Root Mean Square Error (RMSE), which is obtained by:

$$RMSE=\sqrt{\frac{1}{n}\,\mathop{\sum }\limits_{i=1}^{n}\,{({y}_{i}-{\hat{y}}_{i})}^{2}}$$

where *y* is the observed value and $\hat{y}$ is the predicted value by the model.

In the hold-out method, the datasets are randomized, and usually, 2/3 of the data is used for the training of the model, and the remaining 1/3 is used for the model evaluation. In our study, each model was trained and evaluated 30 times, allowing a more confident statistical evaluation. The initial evaluation aimed to determine the most suitable RNN topology for our data. The data were organized into a table consisting of 5 columns, the first four being the RNN inputs (temperature, barometric pressure, solar irradiance, previous activity), and the fifth the expected output in hours’ time (see Fig. 3).

As previously mentioned, as a first step we seek to exploit several RNN architectures. After that, we evaluate how different input size windows impact on the accuracy of the forecast. Finally, we show how algorithms of attribute selection and correlation analysis can help in improving even further the accuracy of the forecast.

### Finding the Best Size for the Input Window

Aiming to advance the results of the forecast, a second evaluation was performed. We employ the best architecture found in the previous step and evaluate different sizes for the input window, that is, different amounts of preceding data to forecast the next level. It demands the RNN the ability to keep valuable information through time. The evaluations were undertaken to employ the current hour, 3, 6, 12, 24, 36, 48, and 60 hours prior to the event.

**Figure 4:**
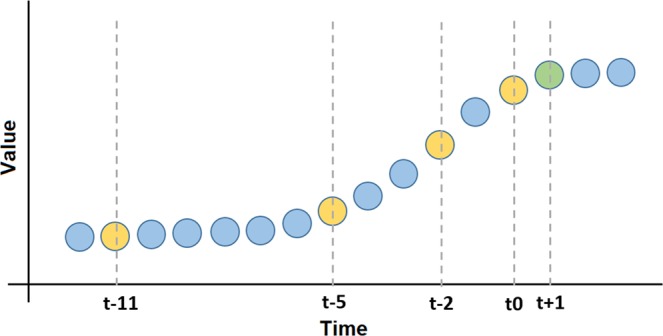
Schematic diagram presenting generic inputs and outputs values to be used in the forecast model. The points in orange represent the values to be employed as inputs of the RNN. The points in green represent output values. Note that this is a generic time series and it is not intended to directly represent real data.

Henceforth, we represent current hour as *t*_0_ and 3, 6, 12, 24, 36, 48 and 60 hours as *t*_−2_, *t*_−5_, *t*_−11_, *t*_−23_, *t*_−35_, *t*_−47_, *t*_−59_ (see Table 1 and Fig. 4). We perform the same test using bees’ level of activity and environmental variables (solar irradiance, barometric pressure and temperature). Table 1 shows the evaluated input vectors (for bees’ level of activity, barometric pressure, solar irradiance and temperature).

**Table 1:** Structure of the evaluated input vectors (for bees’ level of activity, barometric pressure, solar irradiance and temperature).

Reference	Used Values (Input Vector)	Forecast
*w*1	*t*_0_	*t*_+1_
*w*3	*t*_0_, *t*_−2_	*t*_+1_
*w*6	*t*_0_, *t*_−2_, *t*_−5_	*t*_+1_
*w*12	*t*_0_, *t*_−2_, *t*_−5_, *t*_−11_	*t*_+1_
*w*24	*t*_0_, *t*_−2_, *t*_−5_, *t*_−11_, *t*_−23_	*t*_+1_
*w*36	*t*_0_, *t*_−2_, *t*_−5_, *t*_−11_, *t*_−23_, *t*_−35_	*t*_+1_
*w*48	*t*_0_, *t*_−2_, *t*_−5_, *t*_−11_, *t*_−23_, *t*_−35_, *t*_−47_	*t*_+1_
*w*60	*t*_0_, *t*_−2_, *t*_−5_, *t*_−11_, *t*_−23_, *t*_−35_, *t*_−47_, *t*_−59_	*t*_+1_

Related to the input vector, *t*_0_ means the current time (i.e. the hour before the event to forecast), *t*_−2_ means 3 hours before the event to forecast, *t*_−5_ means 6 hours before the event to forecast an so on. Figure 4 shows a graphical representation of the input vector.

Figure 4 presents a schematic diagram presenting generic inputs and outputs values to be used in the forecast model. The figure presents the model we use, although, it does not represent real data gathered by the system. The aim is intended to graphically represent the mean in winch the values are employed. In this figure, we represent the window *w*12 which is a vector composed by the current time (*t*_0_), 3, 6 and 12 hours prior the event to forecast.

### Selecting the Best Environmental Features

Our third effort to improve the forecast accuracy was performed using a technique to select the best environmental predictors. This process consists of selecting the best time window for each variable, join them in one dataset and select the most important ones with lower temporal correlation. As Table 1 shows, each window can have a maximum of 8 temporal values. It means that, a dataset incorporating all 3 environmental variables could have 24 features. For this reason, we selected the best features based on feature importance score and correlation values.

**Figure 5:**
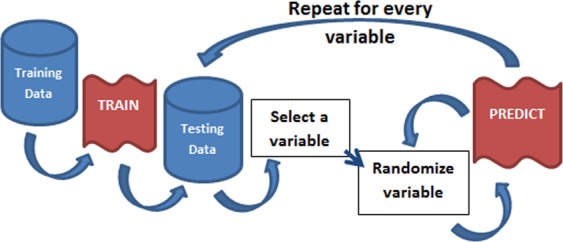
Feature Importance algorithm.

In order to calculate the feature importance score, we used the Permutation Feature Importance (PFI) method. This algorithm works as shown in Fig. 5. After it shuffles a variable, it allows the verification of the new value of RMSE, guiding the process of removing unnecessary or disturbing features. The PFI works by fitting the RNN with the training set and then applying this RNN to the test data (*Dt*). The RMSE found is called *Eo*. Each input feature is shuffled on the corresponding column *Dt* generating *Dt*′. Applying the RNN on this *Dt*′ will give us a new RMSE called *Ed*. The difference between *Ed* and *Eo* is called the feature importance score of the “suffled” feature. A high score means that *Ed* is bigger than *Eo*, in other words, removing the particular feature increases the model’s RMSE.

## Results

### Investigation on RNN architectures

**Figure 6:**
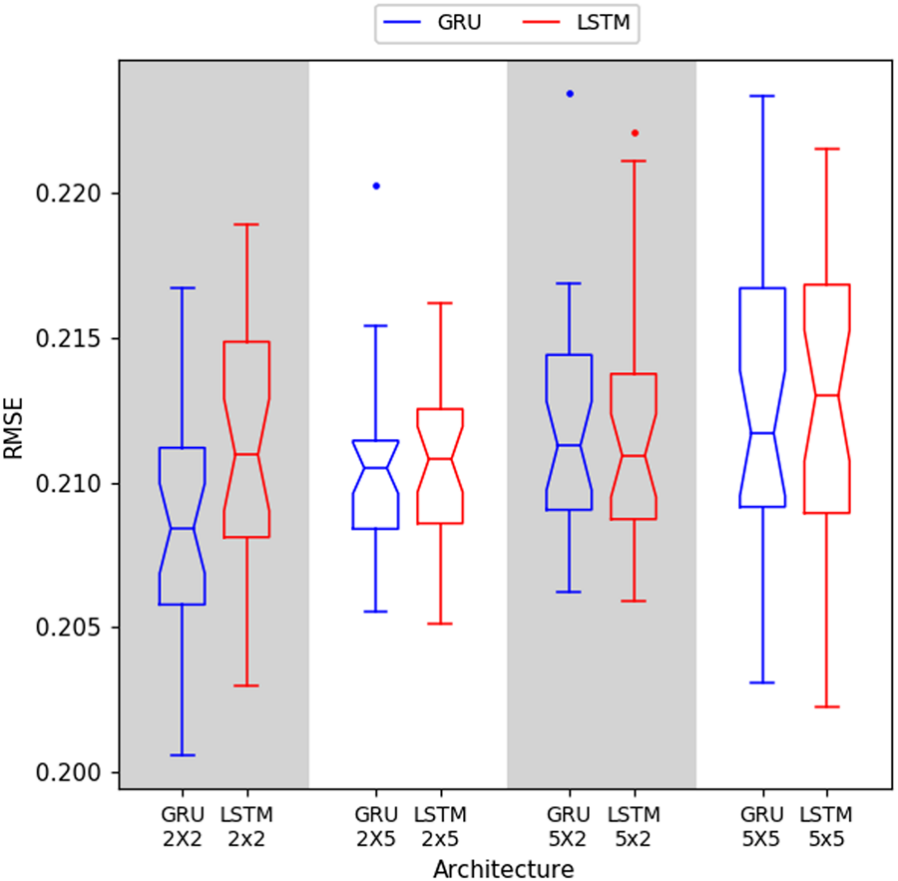
Result (RMSE) for each architecture. A Welch Two Sample t-test showed that the GRU2x2 RNN outperforms the other topologies. The GRU2x2 RNN shows a mean RMSE of 0.208.

Our first evaluation aims to understand which is the best topology to solve our forecast problem. We take into account the current level of activity (act_*t*0_) and environmental features such as temperature (temp_*t*0_), solar irradiance (rad_*t*0_), and barometric pressure (press_*t*0_) to forecast the next level of activity (act_*t*+1_). Due to the random initialization of the network’s weights, each architecture was trained and evaluated 30 times. Figure 6 presents the error (RMSE) for each architecture.

In order to validate which RNN architecture best suited this context, the 8 RNNs were statistically compared. First, we used the Shapiro-Wilk normality test to verify the distribution of the results. For all the sets, except GRU2x5, the Shapiro-Wilk showed p-values larger than 0.05, which means that the distributions can be accepted as parametric ones (i.e. Gaussian distributions). Hence, we employed the Welch Two Sample t-test to determine the similarity among the results. Since the GRU2x2 showed the lowest median (RMSE = 0.208), we compared it to the others. The comparison of GRU2x2 (lowest median) with other architectures showed p-values smaller than 0.05. Thus, GRU2x2 is the most appropriate architecture for the proposed context.

### Investigation on the input window size of bees’ level of activity and weather attributes

Our second evaluation aims to determine the best size for the input window. Input attributes were analyzed individually in order to find the best temporal window size for each one. Hence, we employed the best topology found in the previous evaluation with different windows size (input vectors *w*1 to *w*60, as shown in Table 1). We took into account the following inputs: bees’ level of activity, temperature, solar irradiance and barometric pressure.

**Figure 7:**
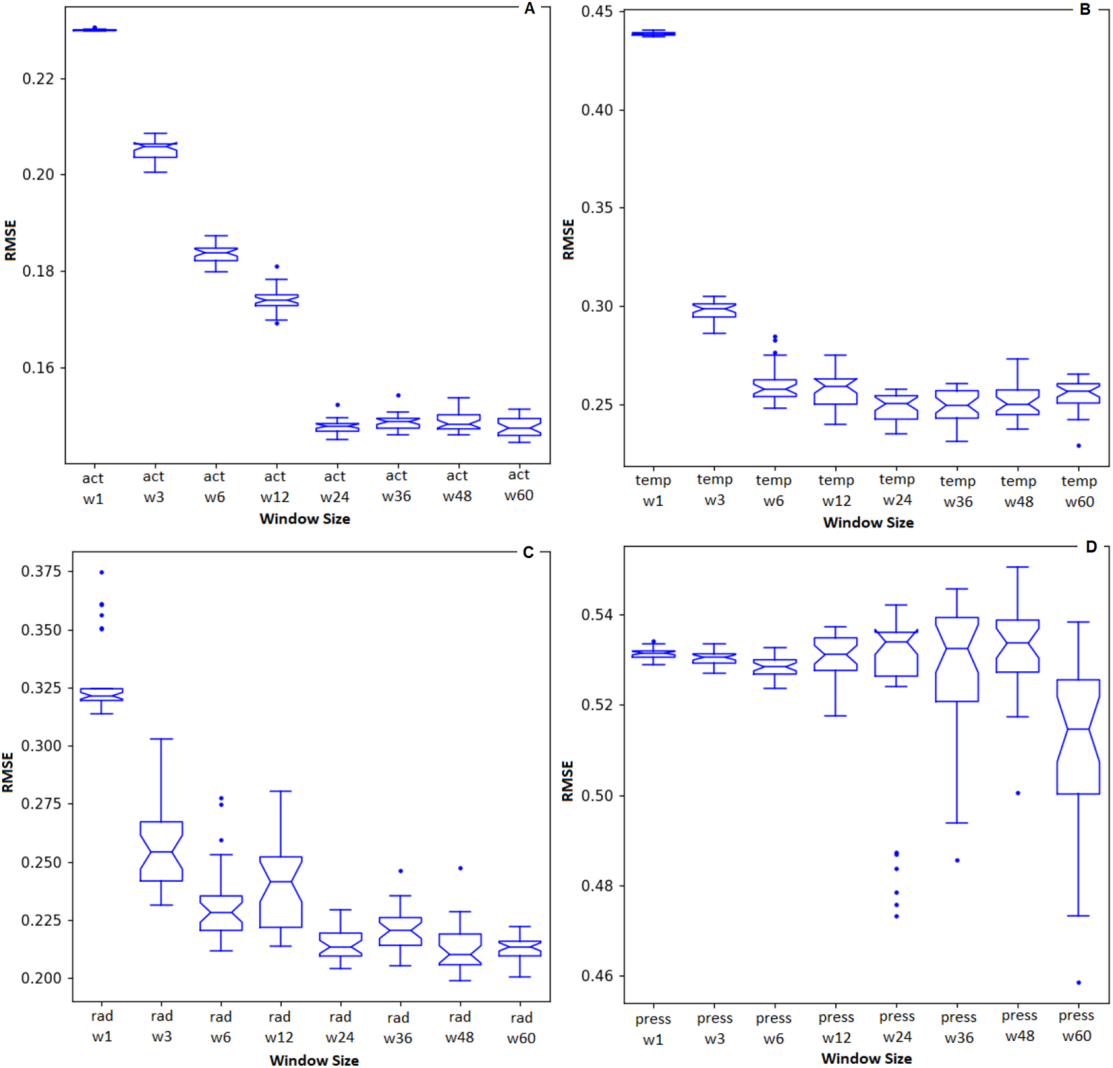
RMSE for each evaluated window size. (**a**) Activity. (**b**) Temperature. (**c**) Solar Irradiance. (**d**) Barometric Pressure.

Figure 7 shows the errors for each different input vector. Results are presented from 30 executions of each RNN. Figure 7(a) shows the error taking into account different window size of preceding level of activity forecasting next levels of activity. The best (lowest) median value was found in the set Act_*w*60_ with an RMSE of 0.147. We performed a statistical test among the sets to verify if any other set is equivalent to the Act_*w*60_. The Welch Two Sample t-test between Act_*w*60_ and Act_*w*24_ showed a p-value of 0.90. Hence, the sets can be considered as equivalent with a confidence level of 95%. Taking into account that the set Act_*w*24_ used fewer variables and the results were statistically equivalent to Act_*w*60_, we chose Act_*w*24_ as the best set of attributes *level of activity* forecasting next levels of activity.

Figure 7(b) shows the error taking into account different window size of temperature forecasting levels of activity. The best (lowest) median value was found in the set Temp_*w*36_ with an RMSE of 0.249. We performed a statistical test among the sets to verify if any other set was equivalent to the Temp_*w*36_. The Welch Two Sample t-test between Temp_*w*36_ and Temp_*w*24_ showed a p-value of 0.60. Hence, the sets can be considered as equivalent with a confidence level of 95%. Taking into account that the set Temp_*w*24_ used fewer variables and that the results were statistically equivalent to Temp_*w*36_, we chose Temp_*w*24_ as the best set for the attribute *temperature* forecasting next levels of activity. Figure 7(c) shows the error taking into account different window size of solar irradiance forecasting levels of activity. The best (lowest) median valeu was found in the set Rad_*w*48_ with an RMSE of 0.210. We performed a statistical test among the sets to verify if any other set was equivalent to the Rad_*w*48_. The Welch Two Sample t-test between Rad_*w*48_ and Rad_*w*24_ showed a p-value of 0.52. Hence, the sets can be considered as equivalent with a confidence level of 95%. Taking into account that the set Rad_*w*24_ used fewer variables and that the results were statistically equivalent to Rad_*w*48_, we chose Rad_*w*24_ as the best set for the attribute *solar irradiance* forecasting levels of activity.

Finally, Fig. 7(d) shows the error taking into account different window size of barometric pressure forecasting levels of activity. The best (lowest) median was found in the set Press_*w*60_ with an RMSE of 0.514. We performed a statistical test among the sets to verify if any other set was equivalent to the Press_*w*60_. No other set showed statistical similarity, being all the comparisons presenting p-values lower than 0.05. Hence, the sets can be considered distinct with a confidence level of 95%. Taking into account that the set Rad_*w*60_ showed the best median value and no other set was equivalent, we chose Press_*w*60_ as the best set for the attribute *barometric pressure* forecasting levels of activity.

The next section presents a combination of the best sets of activity and weather variables obtained in this evaluation, seeking to improve accuracy.

#### Combining Features

After determining the best window size for each attribute, we extended the analysis by testing various combinations of attribute predictors. Thus, the following sets were created: {Activity (A)}, {Activity, Solar Irradiance, Temperature (ART)}, {Activity, Solar Irradiance, Temperature, Barometric Pressure (ARTP)}, {Solar Irradiance, Temperature (RT)}, {Solar Irradiance, Temperature, Barometric Pressure (RTP)}.

For theses sets, we employed the best input size windows found in the previous evaluation: activity, solar irradiance and temperature using window = 24 and barometric pressure using window = 60. We evaluate sets with both previous activity (sets with A) or not (sets without A), since the data from activity may not always be available – in this case, we can estimate the level of activity using weather variables alone. The main motivation in using environmental variables to forecast bees’ activities is motivated by the fact that the RFID system is not always available for use due to the cost and/or management aspects.

**Figure 8:**
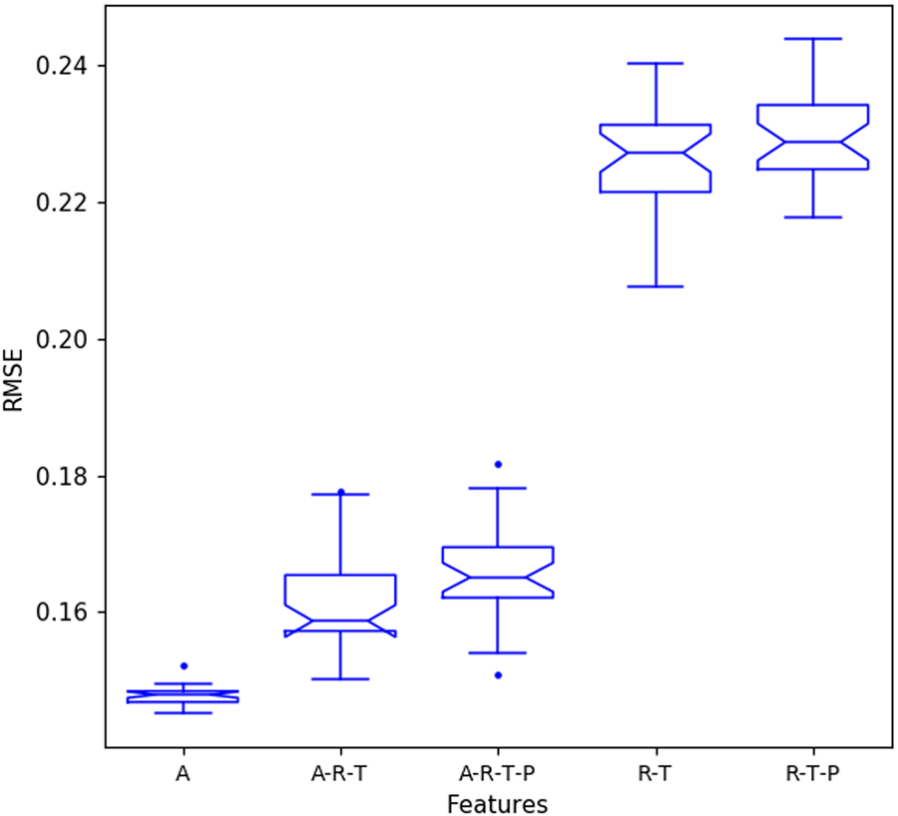
Result (RMSE) for the feature combination incorporating the best window size for each attribute.

Figure 8 shows the result (RMSE) for the feature combination with the best windows size of each attribute. We can see that using previous activity (w24) alone is the best to forecast next levels. Although it seems that the sets in which barometric pressure is used, have decreased accuracy, a statistical comparison among RT and RTP shows that they are statistically equivalent.

As previously defined, the activity level is calculated considering the total number of bees’ activities divided by the number of live bees at that period. Which means that, knowing the activity levels of the preceding 24 hours, we can forecast the level of activity with an average RMSE of 0.147. Since the activity level ranged from 0.0 to 2.0, the mean error of this configuration is about 8%. Taking into account that the average error in the first evaluation (GRU2x2 ARTP_w1) was 0.208 and the average error using the ACT_w24 is 0.147, we have about 30% improvement in the accuracy by using ACT_w24 window size as input.

In the next section we exploit the Permutation Feature Importance algorithm and perform a Correlation Analysis aiming to improve the forecast accuracy, employing weather variables alone.

### Feature importance and correlation analisys

We aim to investigate the importance of environmental variables alone as the predictors for bees’ activity level, employing the following environmental variables: solar irradiance (R), temperature (T) and barometric pressure (P). Therefore, we exploit the Permutation Feature Importance algorithm and perform a Correlation Analysis. We took into account the best sets found in previous section: Solar Irradiance and Temperature with w24 and Barometric Pressure with w60.

**Figure 9:**
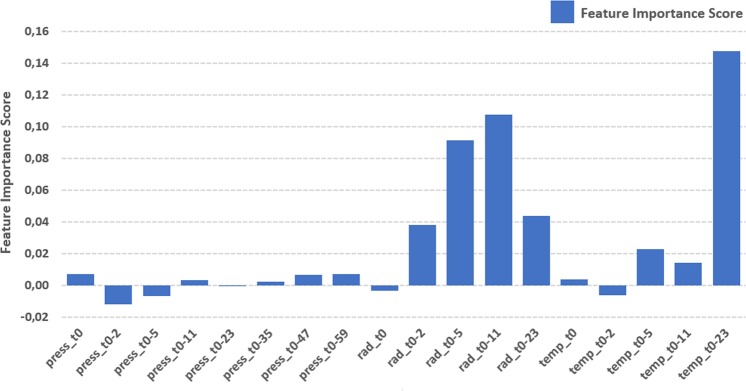
Feature Importance score for each attribute. Higher the score, higher the influence to improve model’s accuracy.

**Figure 10:**
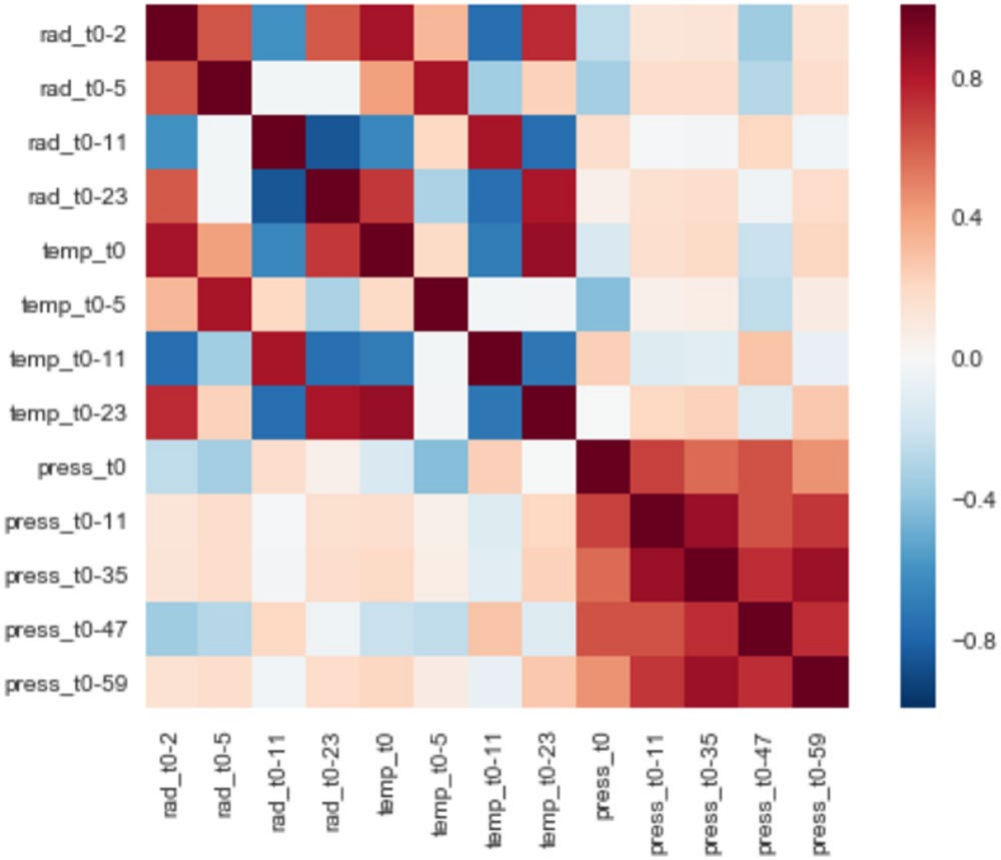
Feature correlation heatmap.

Figure 9 shows the feature importance score for each attribute. We can see that temperature and solar irradiance present higher scores, which suggests a strong influence on bees’ activity level. Furthermore, we can see some attributes with lower than zero score, which suggests that they are decreasing model’s accuracy. Figure 10 shows the feature correlation heatmap. Highly correlated attributes are often considered redundant because they do not add useful information to the model. Furthermore, they can add noise and be a confounding factor in the training of models. Hence it is a good practice to remove highly correlated attributes.

We created 3 new datasets based on the results of feature importance (scores) and correlation values. The first considers all features with score larger than 0.0 (named FSL0). We then evaluated the correlation among the attributes, and created datasets removing attributes that showed correlation larger than 70% and 80% (named CORR70 and CORR80 [upon FSL0]). We aim to evaluate if the accuracy improves when removing highly correlated attributes, given that highly correlated attributes may be a confounding factor when used in conjunction.

**Figure 11:**
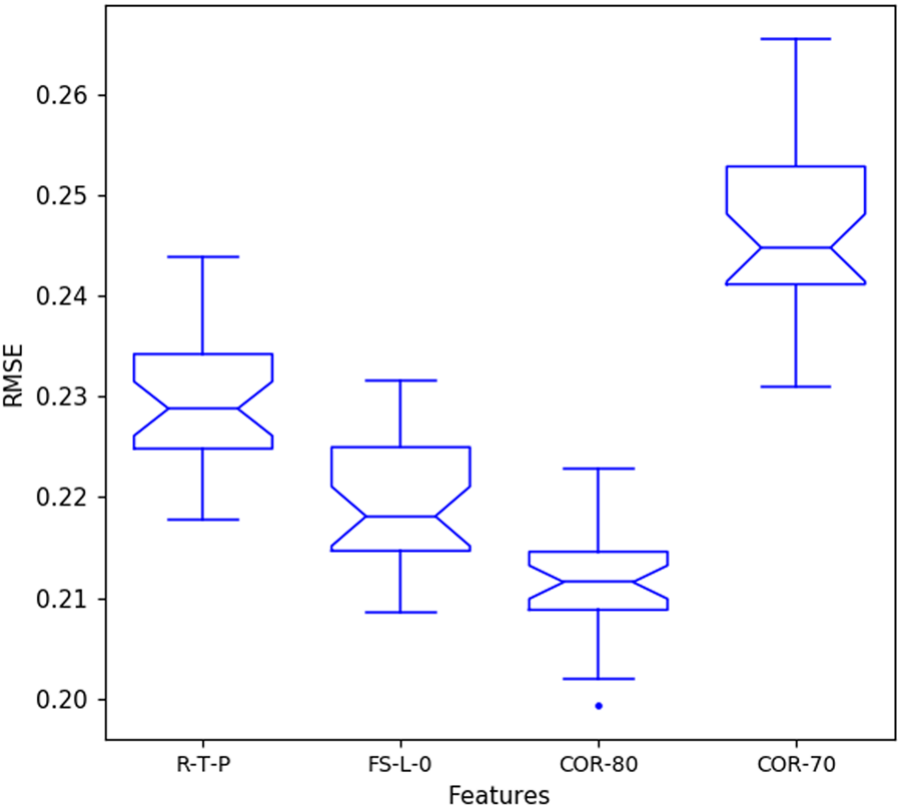
Results using sets with feature score larger than 0 (FSL0), sets removing correlated attributes (correlation greater than 80% and 70%) and sets with the best window size of RTP found in previous section (R_w24, T_w24, P_w60).

Figure 11 shows the result of the RNN when using sets with feature score larger than 0 (FSL0), sets removing correlated attributes (correlation greater than 80% and 70%), and also shows the RTP found in the evaluation of window size (R_w24, T_w24, P_w60). We used the Shapiro-Wilk normality test to verify the adequacy of the results to parametric or non-parametric distributions. For all the sets the Shapiro-Wilk showed p-values larger than 0.05, which means that the distributions can be accepted as parametric ones. Hence, we employed the Welch Two Sample t-test to verify the similarity among the results. The comparison showed that both sets are distinct from each other, since all tests showed p-value smaller than 0.05 – It means that the results are statistically distinct with 95% of confidence. The best one is the CORR80 since it showed the lowest error.

We can see that the PFI outperforms the regular RTP since it can remove features that have a confounding effect upon model’s accuracy. Moreover, we can see that the mapping results of correlation analysis also demonstrated that a correlation threshold of 80% was ideal in our experiment, however, it must be highlighted that this value is likely problem dependent.

**Figure 12:**
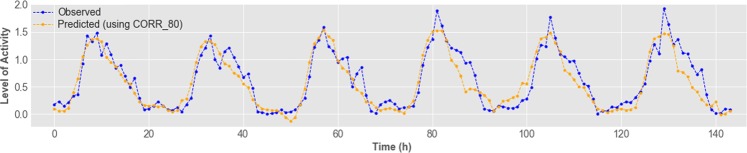
Observed and predicted activity levels, using attributes with lower than 80% correlation and higher than zero permutation feature importance score.

Employing weather variables alone, and using a technique to find the best window size, allowed us to obtain an average RMSE error of 0.229 (Section “Investigation on the Input Window Size of Bees’ Level of Activity and Weather Attributes”). After employing the PFI and performing an analysis of correlated variables, we were able to decrease the average RMSE error to 0.212, being approximately 7.5% better. Figure 12 shows a subset of six days of our data, presenting observed and predicted values, using environmental attributes as predictors.

## Conclusion and Future Work

This work aimed to investigate RNN on the task of predicting bees’ level of activity, which can be approached as a time-series forecasting problem. In the first step, we investigated eight different RNN upon data from bees’ activity and environmental data (temperature, solar irradiance and barometric pressure) finding that GRU outperforms LSTM in this particular problem. It was followed by the evaluation of the best window size for each attribute, in which we perceive that employing larger inputs help improving the accuracy of the model. For example, knowing the activity levels of the preceding 24 hours allowed us to forecast the level of activity with an average RMSE of 0.147, being about 30% better than using only one hour ahead attributes.

In the final step, we exploited the Permutation Feature Importance algorithm and performed a Correlation Analysis aiming to improve the forecast accuracy employing environmental variables alone. Based on the assumption mentioned before, the cost and/or technical aspects could make the RFID system unavailable. For this reason, we investigated the importance of as predictors for bees’ activity level, employing the following environmental variables: solar irradiance (R), temperature (T) and barometric pressure (P). Employing weather variables alone, and using a technique to find the best window size, allowed us to obtain an average RMSE error of 0.229. After employing the PFI and an analysis of correlated variables, we were able to decrease the average RMSE error to 0.212, being approximately 7.5% better.

A better understanding of bees’ behavior can contribute to the environment, fruit producers and to our lives. In this research, we pointed out a way to improve forecast of bees’ activity by means of RNNs. Although, there are some future work we plan to tackle in the continuity of this project; those are more related to the environmental evaluation and the influence of (i) parasites, (ii) pesticides, (iii) weather changes, (iv) monoculture farming, and (v) inappropriate management of beehives.

## Data Availability

The data we use in this study is available at 10.13140/RG.2.2.14287.02723. A sample source-code can be found at 10.13140/RG.2.2.27938.17603.
